# Association between urinary exposures and the risk of chronic obstructive pulmonary disease in smokers: results from NHANES 2007–2016

**DOI:** 10.3389/fpubh.2025.1548401

**Published:** 2025-04-04

**Authors:** Hongli Xu, Weiwei Chen, Jinjun Sun

**Affiliations:** Department of Tuberculosis, Affiliated Hospital of Shaoxing University, Zhejiang, China

**Keywords:** exposures, COPD, NHANES, mediation analysis, inflammatory markers

## Abstract

**Objective:**

This study aims to shed light on the connection of urinary exposures with risk of chronic obstructive pulmonary disease (COPD) among smokers, thereby providing scientific evidence for the prevention and intervention of COPD.

**Methods:**

Data of the National Health and Nutrition Examination Survey (NHANES) 2007–2016 were utilized, including 3,973 smokers aged 20 or older. We employed the weighted multivariate logistic and weighted quantile sum (WQS) regression models to delve into the link of urinary concentrations of exposures to COPD risk. Additionally, restricted cubic spline regression was utilized to examine the dose–response relationship between biomarker concentrations and COPD risk. The stability of the associations across different participant characteristics was evaluated through subgroup and mediation analyses.

**Results:**

Our study encompassed a total of 3,973 participants, of whom 472 were diagnosed with COPD. Regression analyses revealed the inverse association between urinary concentrations of benzophenone-3 (BP-3) and propyl paraben (PrP) and COPD risk. Higher quartiles of BP-3 and PrP exhibited lower COPD incidence [BP-3: odds ratio (OR) = 0.64, 95% confidence interval (95%CI) (0.47, 0.89), *p* = 0.007; PrP: OR = 0.56, 95%CI (0.36, 0.86), *p* = 0.008]. Significant synergistic interactions among urinary exposures were observed [WQS: 0.75, 95%CI (0.65, 0.88), *p* = 0.026], with BP-3 and PrP contributing 40.31 and 40.01% to the weighted analysis, respectively. Mediation analysis proved that inflammatory markers, such as white blood cell (WBC) count and neutrophil-to-lymphocyte ratio (NLR), significantly mediated the association between BP-3, PrP, and COPD risk (all *p*-values <0.05).

**Conclusion:**

BP-3 and PrP in environmental exposure in smokers have an inverse correlation with COPD risk, with WBC and NLR partially mediating this association.

## 1 Introduction

Chronic obstructive pulmonary disease (COPD) is a common respiratory disorder due to long-term exposure to harmful particles or gases, leading to complications such as cor pulmonale and respiratory failure (1). According to the World Health Organization, COPD is the third major cause of mortality globally, and imposed an annual economic burden exceeding $100 billion (2). The onset and progression of COPD are influenced by an interplay between host susceptibility factors and various risk factors (3). Smoking is the most prevailing risk factor for COPD worldwide, with studies showing that smokers have a 10.92-fold higher risk of developing COPD than non-smokers (4, 5). In comparison to non-smoking COPD patients, smokers with COPD exhibit more respiratory symptoms, an increased incidence of pulmonary function abnormalities, and a faster reduction in forced expiratory volume in 1 s (FEV1), which were positively correlated with cumulative smoking exposure (6). Although smoking is a major contributor to COPD, non-tobacco risk factors account for over 50% of cases worldwide. Besides smoking, exposure to environmental pollutants has a primary role in incidence and development of COPD (3, 7). Therefore, it is necessary to identify environmental pollutants linked to COPD risk, thereby preventing this long-lasting illness.

Recent research has focused on the detrimental effects of endocrine-disrupting chemicals (EDCs) like phenols and parabens, on lung function (8). Phenols, parabens, and phthalates, present broadly in food packaging, drugs, and construction materials, easily enter the environment (e.g., air, dust, food, and water) and human body through inhalation, ingestion, and dermal absorption because of their chemical properties (9). More evidence supports the correlation between EDC exposure and adverse respiratory outcomes in adults (10–12). Several studies have proved that EDCs can influence the development, function, and lifespan of immune cells, such as monocytes, neutrophils, eosinophils, and lymphocytes, and potentially impair the function of lung (13, 14). Associations have been found between decreased FEV1 and exposure to various phenols, parabens, and five phthalate metabolites (15). Specifically, bisphenol A (BPA) has been demonstrated to inhibit neutrophil chemotaxis *in vitro*, and environmental exposure to BPA can lead to lung inflammation, dysfunction, and impaired lung maturation (16, 17). In animal models, urinary 4-vinylphenol (4-VP) has been implicated in styrene-induced cytotoxicity with marked pulmonary toxicity (18). Prolonged di-(2-ethylhexyl) phthalate (DEHP) exposure in mice results in eosinophil recruitment, thereby promoting allergic lung inflammation (19, 20), while butyl benzyl phthalate (BBP) treatment in mice causes considerable lung injury featuring alveolar hemorrhage, pulmonary edema, as well as heightened neutrophil infiltration (21). However, it has been suggested that these EDCs may impact varied pulmonary function parameters in varying ways (22), and the effects of different doses of EDCs may vary (15). For example, BPA exposure is linked to a reduction in forced expiratory flow at 25 to 75% of forced vital capacity (FEF_25-75%_) but not to FEV1 or forced vital capacity (FVC) (22). Buckley et al. (23) noted no link of low-molecular-weight phthalate concentrations to asthma or eczema, whereas Vernet et al. (24) reported an association between parabens and asthma or wheezing, though Lee-Sarwar et al. (25) identified no such relation to asthma, wheeze, or aeroallergen sensitization. To further elucidate the correlation between exposure to these chemicals and the risk of COPD in smokers, we obtained data from the National Health and Nutrition Examination Survey (NHANES) 2007–2016 to unravel the connection of environmental exposure to phenols and parabens with COPD risk in smokers, thereby providing scientific evidence for COPD prevention and intervention.

## 2 Methods

### 2.1 Data source

NHANES, a representative survey in America, seeks to assess the health and nutrition of the non-institutionalized Americans, and features classified sampling in multiple stages, including interview, physical examination, and laboratory test. We mainly extracted COPD-related data of patients who are 20 or older from 2007 to 2016. The NHANES protocol received approval from the National Center for Health Statistics at the Centers for Disease Control and Prevention, with participants providing informed consent upon enrollment. All data are accessible at http://www.cdc.gov/nchs/nhanes/about_nhanes.htm. The Strengthening the Reporting of Observational Studies in Epidemiology (STROBE) guidelines was followed.

### 2.2 Study population

**Figure 1:**
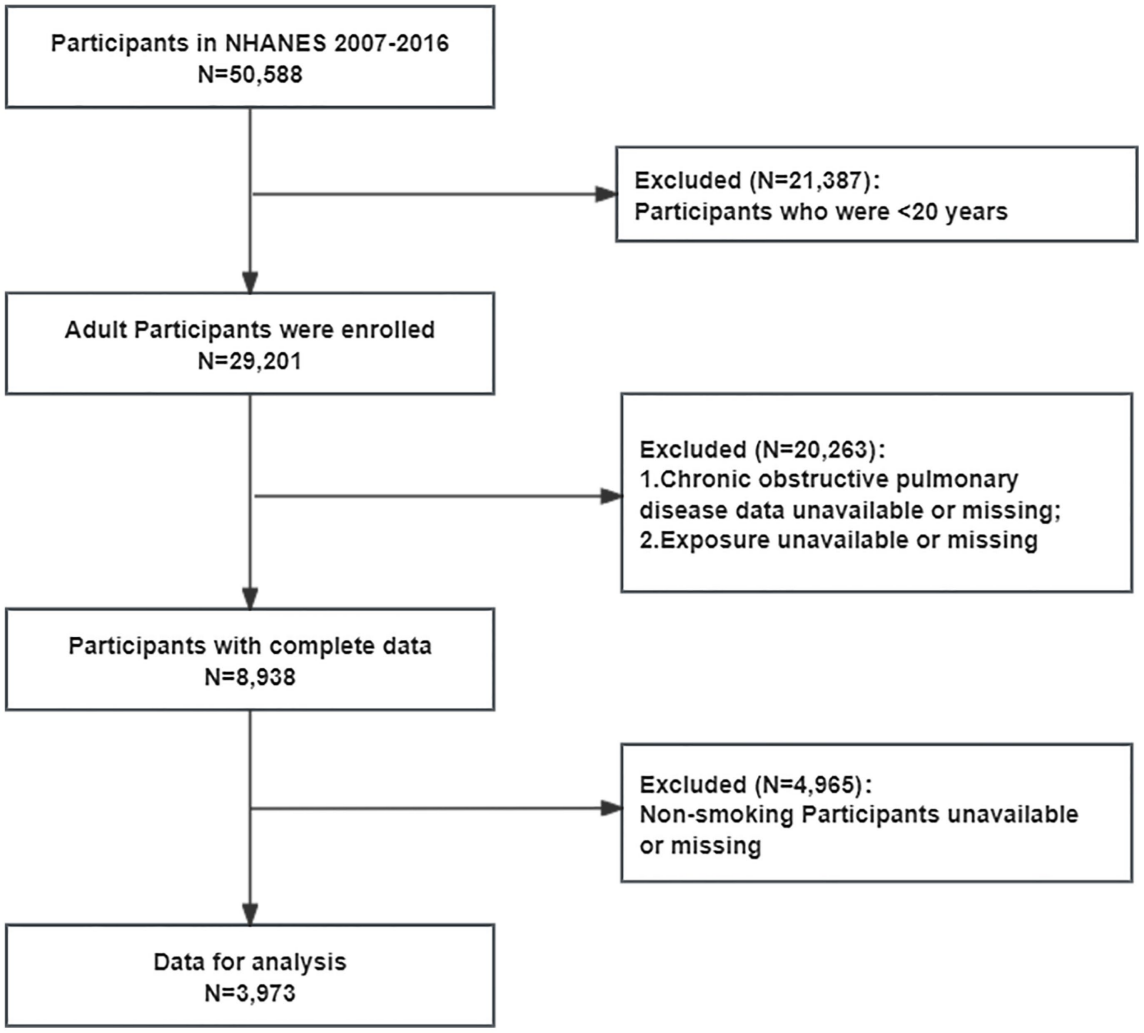
Flowchart of the selection process for eligible participants.

A total of 50,588 individuals from the NHANES were initially screened. Exclusion criteria: (1) age under 20 years; (2) incomplete COPD data; (3) incomplete exposure data; (4) non-smoking population. Inclusion criteria: (1) age ≥ 20 years; (2) complete COPD and exposure data; (3) smoking population. After excluding participants who fail to meet the inclusion criteria, 3,973 participants was ultimately included in our study, representing approximately 120 million individuals. The screening process is presented in Figure 1.

### 2.3 Assessment of exposures

Seven chemicals were measured in the urine of the study participants: benzophenone-3 (BP-3), BPA, triclosan (TCS), butyl paraben (BuP), ethyl paraben (EtP), methyl paraben (MeP), and propyl paraben (PrP). Their respective limits of detection (LOD) were 0.4 ng/mL, 0.2 ng/mL, 1.7 ng/mL, 0.1 ng/mL, 1.0 ng/mL, 1.0 ng/mL, and 0.1 ng/mL. We substituted concentrations under LOD with the square root of LOD divided by 2 (LOD/√2) [Measurement details: https://wwwn.cdc.gov/Nchs/Nhanes/2013-2014/EPHPP_H.htm#URXEPB].

### 2.4 Assessment of COPD and smoking population

Assessment of COPD and smoking population were based on questionnaire data. COPD patients were those answering yes to the question, “Has you been told by a doctor or other health professional that you have COPD/emphysema/chronic bronchitis” Participants were categorized into three groups in terms of their smoking status: never smokers (who had smoked fewer than 100 cigarettes in their lifetime), former smokers (who had smoked more than 100 cigarettes in their lifetime but were not currently smoking), and current smokers (who had smoked over 100 cigarettes in their lifetime and reported smoking on some days or each day at the time of interview). For this study, we included only former and current smokers as our study population.

### 2.5 Assessment of covariates

Covariates included demographic and health-related data such as age (>50, 50–60, 60–70, ≥70 years), gender (male or female), race (Mexican, white, black, other), marital status (married, unmarried, other), education level (less than high school: high school includes 9th grade, 9-11th grade [including 12th grade with no diploma], High school or equivale, college graduate or above), drinking (non-drinkers, Low to moderate drinkers had <1 drink per day for women and < 2 drinks per day for men, heavy drinkers drink once or more times daily for women and two mor more times every day for men), and Poverty-to-Income Ratio (PIR) (<=1.0, 1.1-3.0, > 3.0), physical activity (yes, no), body mass index (BMI) derived through weight (kg)/height (m)^2^. Hypertension was identified according to responses to questions such as, “{Have you/Has SP} been told by a doctor or other health professional that {you/s/he} had hypertension, or high blood pressure?” or “Because of {your/SP’s} (high blood pressure/hypertension), {have you/has s/he} been told to take prescribed medicine?” or the diagnostic criteria for hypertension included a diagnosis of hypertension from a doctor or use of antihypertensive medication, or a systolic blood pressure over 140 mmHg or diastolic pressure over 90 mmHg. This variable was classified into two groups: yes and no. Regarding diabetes, participants were determined as those (1) diagnosed with diabetes during the survey; (2) with HbA1c ≥ 6.5% classified as diabetic; (3) taking diabetes medication to lower blood sugar; (4) currently taking insulin. The University of Minnesota Laboratory and the University of Missouri-Columbia examined other biochemical measurements.

### 2.6 Data analysis

Analyses were conducted with the help of R (version 4.3.1) with a complicated sampling design of NHANES. Given the complex probability clustering design of NHANES, we considered sample weights in all statistical analyses. We combined five 2-year cycles of NHANES data. The new weight was obtained by dividing the original 2-year sample weight by 5, following NHANES weighting guidelines. Log-transformation of the exposure concentrations was performed to standardize their distribution for further analysis. We classified environmental exposures into quartiles, with Q1 serving as reference. Weighted multivariable-adjusted logistic regression models were utilized in R, with statistical significance defined as *p* < 0.05. Results were displayed as odds ratios (OR) with 95% confidence intervals (CI). We employed restricted cubic spline (RCS) plots to investigate the non-linear and dose–response relationships between environmental exposures and COPD. The weighted quantile sum (WQS) regression model, a statistical one employing multivariate regression on high-dimensional datasets, assisted in assessing the association between environmental exposures and COPD and calculating respective weights. Through a weighted index, it created a composite outcome variable from multiple exposure variables, and assigned weights to every variable for assessing the relative importance of individual variables to the outcome. Additionally, subgroup analyses helped to examine the influence of age, gender, race, marital status, education level, physical activity and drinking, Poverty-to-Income Ratio (PIR), BMI, diabetes, as well as hypertension on the link of environmental exposure concentrations to COPD prevalence. Interaction effects were evaluated by adding multiplicative interaction terms between environmental exposures and corresponding stratifying variables.

The potential mediating effects of white blood cell (WBC), neutrophil-to-lymphocyte ratio (NLR), and systemic immune-inflammation index (SII) on the relationship between exposures and COPD risk were evaluated via mediation models. We performed mediation analysis through a quasi-Bayesian Monte Carlo method and a thousand simulations using the normal approximation. Direct effects (DE) represent the impact of exposure on COPD without the mediator, while indirect effects (IE) denote that through the mediator. The proportion mediated was derived by dividing the IE by the total effect (TE).

## 3 Results

### 3.1 Baseline characteristics of the study population and distribution of exposures

3,973 participants were selected. Table 1 summarized their characteristics based on COPD. Among the smokers, 3,501 did not have COPD, while 472 were diagnosed with COPD. The majority of COPD patients were under 50 years old, with a slightly higher prevalence among females compared with males. Most COPD patients were married, predominantly of white ethnicity, and had a college education or higher. The degree of poverty was assessed through PIR, with a greater number of COPD patients having a PIR between 1.1 and 3.0. Additionally, the COPD population have an elevated BMI (≥30), never participate in physical exercise, suffer from hypertension, consume moderate to low amounts of alcohol, and not have diabetes.

**Table 1:** Weighted baseline characteristics.

Variable	Level	Overall	Non-COPD	COPD	*p*-value
*n*		3,973 (122353618.59)	3,501 (108042707.56)	472 (14310911.03)	
Age, *n*%	< 50	1749 (49.8)	1,613 (52.0)	136 (33.4)	<0.001
	50–60	805 (22.7)	695 (22.1)	110 (27.1)	
	60–70	764 (15.6)	647 (14.7)	117 (22.0)	
	> = 70	655 (11.9)	546 (11.2)	109 (17.5)	
Gender, *n* (%)	Female	1,639 (44.7)	1,387 (43.2)	252 (56.4)	<0.001
	Male	2,334 (55.3)	2,114 (56.8)	220 (43.6)	
Marital status, *n* (%)	Married	2,319 (62.7)	2073 (63.3)	246 (58.4)	<0.001
	Unmarried	650 (16.1)	597 (16.9)	53 (9.7)	
	Other	1,004 (21.2)	831 (19.8)	173 (32.0)	
Race, *n* (%)	White	1961 (72.9)	1,670 (72.1)	291 (79.2)	<0.001
	Black	832 (10.1)	750 (10.4)	82 (7.9)	
	Mexican	498 (6.5)	469 (7.0)	29 (2.1)	
	Other	682 (10.5)	612 (10.5)	70 (10.7)	
Education level, *n* (%)	College graduate or above	1778 (52.7)	1,594 (53.9)	184 (43.3)	<0.001
	High school or Equivale	1,056 (26.9)	936 (26.7)	120 (28.7)	
	Less than high school	1,139 (20.4)	971 (19.4)	168 (28.1)	
PIR, *n* (%)	<= 1.0	1,022 (17.4)	867 (16.5)	155 (24.3)	<0.001
	1.1–3.0	1721 (37.6)	1,504 (36.9)	217 (43.0)	
	> 3.0	1,230 (45.0)	1,130 (46.7)	100 (32.7)	
BMI, *n* (%).	< 18.5	70 (1.6)	50 (1.1)	20 (5.3)	<0.001
	18.5–24.9	1,047 (27.9)	938 (28.5)	109 (23.3)	
	24.9–29.9	1,326 (31.9)	1,201 (32.8)	125 (24.4)	
	> = 30	1,530 (38.6)	1,312 (37.5)	218 (47.0)	
Physical activity, *n* (%)	No	2,454 (57.3)	2,148 (56.8)	306 (61.5)	0.159
	Yes	1,519 (42.7)	1,353 (43.2)	166 (38.5)	
Drinking, *n* (%)	Heavy drinker	536 (15.2)	480 (15.7)	56 (12.0)	<0.001
	Low to moderate drinker	2,840 (73.0)	2,525 (73.2)	315 (71.6)	
	Non-drinker	597 (11.8)	496 (11.2)	101 (16.4)	
Hypertension, *n* (%)	No	2,382 (63.9)	2,172 (65.8)	210 (49.6)	<0.001
	Yes	1,591 (36.1)	1,329 (34.2)	262 (50.4)	
Diabetes, *n* (%)	No	3,244 (86.2)	2,913 (87.8)	331 (73.7)	<0.001
	Yes	729 (13.8)	588 (12.2)	141 (26.3)	

*p*-value was based on *χ*^2^ or analysis of variance or Kruskal-Wallis rank sum test where appropriate; Values represent weighted means (SD) or *N* (weighted %); PIR, Poverty-to-Income Ratio; BMI, Body Mass Index.

The distribution of urinary exposure concentrations among all study participants is detailed in Supplementary Table S1. The detection rates for EtP and BuP were 49.96% and 30.15%, respectively, and these were excluded for further analysis. The detection rates for other chemical exposures exceeded 70%. The correlations between concentrations of various exposures measured in urine were explored via Spearman rank correlation analysis, and Pearson correlation coefficients were derived. We noted a significant connection between MeP and PrP (r = 0.81), while the correlations among other exposures were moderate or weak (*r* < 0.6) (Supplementary Figure S1).

### 3.2 Association between phenols, parabens, and COPD risk

The weighted logistic regression results showed a notable inverse correlation between BP-3, PrP, and COPD risk across all quartile categories (Table 2). In Model 1, which was unadjusted for covariates, the *OR* and 95%CI for BP-3 in the highest quartile (Q4) were 0.58 (0.43–0.78) (*p* < 0.001). After the adjustment for age, gender, race, education level, marital status, and PIR in Model 2, the association remained similar to the unadjusted model [Q4, 0.59 (0.43–0.82), *p* = 0.001]. Model 3, with additional adjustment for drinking, BMI, diabetes, and hypertension, yielded consistent results, thus proving a notable correlation between BP-3 and COPD risk in Q4 [0.64 (0.47–0.89), *p* = 0.007]. Similarly, PrP was also markedly linked to COPD risk, with *OR* in Q4 for the three models being 0.62 (0.41–0.93, *p* = 0.021), 0.53 (0.35–0.82, *p* = 0.004), and 0.56 (0.36–0.86, *p* = 0.008).

**Table 2:** Association between environmental exposures and COPD.

Expose	Q1	Q2	Q3	Q4	p for trend
BP-3					
	1	0.79 (0.61–1.02, *p* = 0.067)	0.68 (0.52–0.89, *p* = 0.005)	0.58 (0.43–0.78, *p* < 0.001)	0.000
	1	0.84 (0.64–1.09, *p* = 0.193)	0.75 (0.56–0.99, *p* = 0.046)	0.59 (0.43–0.82, *p* = 0.001)	0.001
	1	0.86 (0.66–1.13, *p* = 0.286)	0.78 (0.58–1.03, *p* = 0.083)	0.64 (0.47–0.89, *p* = 0.007)	0.004
BPA					
	1	1.04 (0.79–1.37, *p* = 0.772)	0.77 (0.58–1.04, *p* = 0.090)	1.22 (0.93–1.61, *p* = 0.158)	0.000
	1	1.11 (0.84–1.47, *p* = 0.478)	0.85 (0.62–1.15, *p* = 0.282)	1.32 (0.99–1.76, *p* = 0.062)	0.263
	1	1.03 (0.78–1.37, *p* = 0.822)	0.81 (0.60–1.10, *p* = 0.181)	1.24 (0.93–1.66, *p* = 0.144)	0.412
TCS					
	1	0.79 (0.60–1.04, *p* = 0.095)	0.97 (0.74–1.26, *p* = 0.795)	0.91 (0.69–1.20, *p* = 0.502)	0.566
	1	0.88 (0.66–1.16, *p* = 0.359)	1.08 (0.82–1.43, *p* = 0.573)	1.10 (0.83–1.47, *p* = 0.511)	0.325
	1	0.86 (0.65–1.14, *p* = 0.298)	1.11 (0.84–1.47, *p* = 0.446)	1.13 (0.85–1.51, *p* = 0.406)	0.226
MeP					
	1	1.10 (0.82–1.48, *p* = 0.505)	1.14 (0.80–1.62, *p* = 0.461)	1.28 (0.85–1.93, *p* = 0.237)	0.695
	1	1.13 (0.83–1.53, *p* = 0.431)	1.11 (0.77–1.60, *p* = 0.566)	1.28 (0.84–1.96, *p* = 0.248)	0.282
	1	1.17 (0.86–1.59, *p* = 0.310)	1.14 (0.79–1.64, *p* = 0.489)	1.32 (0.86–2.03, *p* = 0.206)	0.233
PrP					
	1	0.73 (0.55–0.97, *p* = 0.030)	0.81 (0.58–1.14, *p* = 0.226)	0.62 (0.41–0.93, *p* = 0.021)	0.274
	1	0.69 (0.51–0.93, *p* = 0.016)	0.74 (0.52–1.05, *p* = 0.092)	0.53 (0.35–0.82, *p* = 0.004)	0.008
	1	0.68 (0.50–0.92, *p* = 0.014)	0.74 (0.52–1.06, *p* = 0.100)	0.56 (0.36–0.86, *p* = 0.008)	0.013

All estimated results were expressed as OR (95% CI); OR, Odds Ratio; CI, Confidence Interval. Model 1: unadjusted for covariates; Model 2: adjusted for age, gender, race, education level, marital status, PIR; Model 3: adjusted for age, gender, race, education level, marital status, PIR, drinking, physical activity, BMI, diabetes, hypertension. BP-3, Benzophenone-3; BPA, Bisphenol A; TCS, Triclosan; MeP, Methyl paraben; PrP, Propyl paraben.

**Figure 2:**
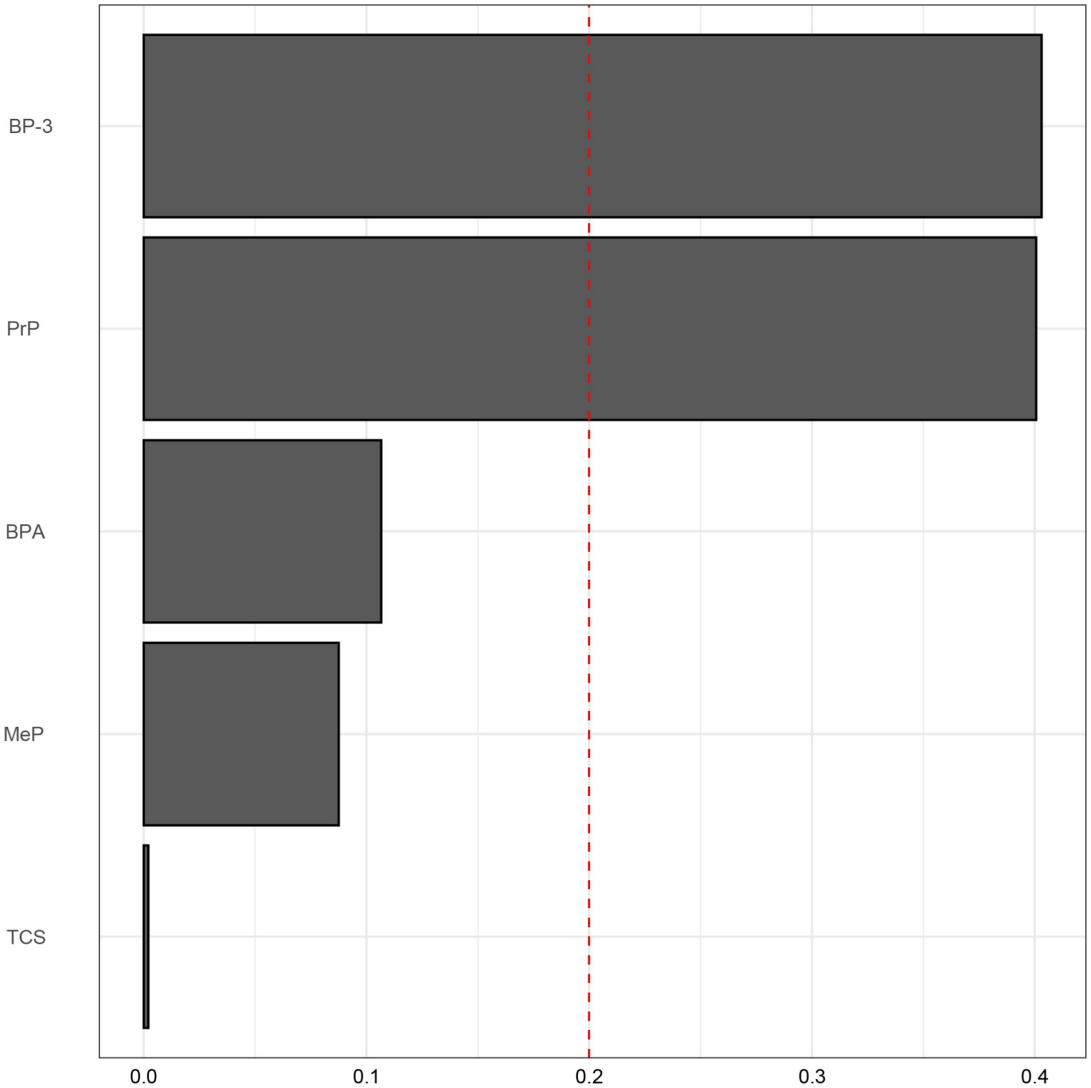
Weighted Values in the WQS Model. The model was adjusted for age, gender, race, education level, marital status, PIR, drinking, physical activity, BMI, diabetes, and hypertension. BP-3, Benzophenone-3; BPA, Bisphenol A; TCS, Triclosan; MeP, Methyl paraben; PrP, Propyl paraben.

**Figure 3:**
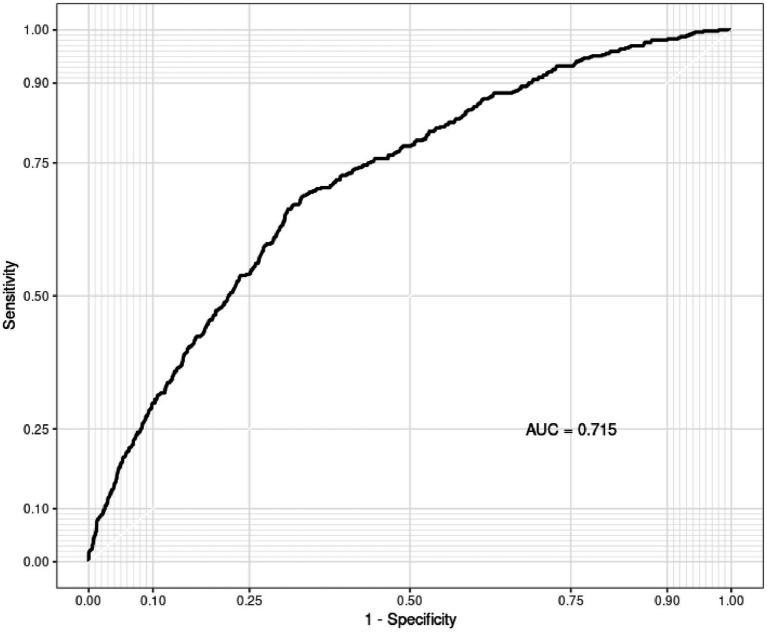
AUC of the WQS Model. The model was adjusted for age, gender, race, education level, marital status, PIR, drinking, physical activity, BMI, diabetes, and hypertension.

To further elucidate the intricate exposure-response relationship between environmental exposures and COPD, the WQS model helped with the assessment of combined effects of environmental exposures on COPD. A WQS index of 0.75 (95%CI: 0.65–0.88, *p* = 0.026), suggested a notable synergistic interaction of varied environmental exposures (see Figure 2). Subsequent analysis on the weight proportions of environmental exposures demonstrated that BP-3 accounted for 40.31%, PrP for 40.01%, while other exposures were below 10%. This indicated that exposure to BP-3 and PrP may have the most substantial impact on COPD risk. The evaluation of the WQS model demonstrated that the area under the ROC curve (AUC) for COPD was 0.715 (0.691–0.739) (Figure 3).

### 3.3 Nonlinear and dose–response relationship between exposures and COPD

**Figure 4:**
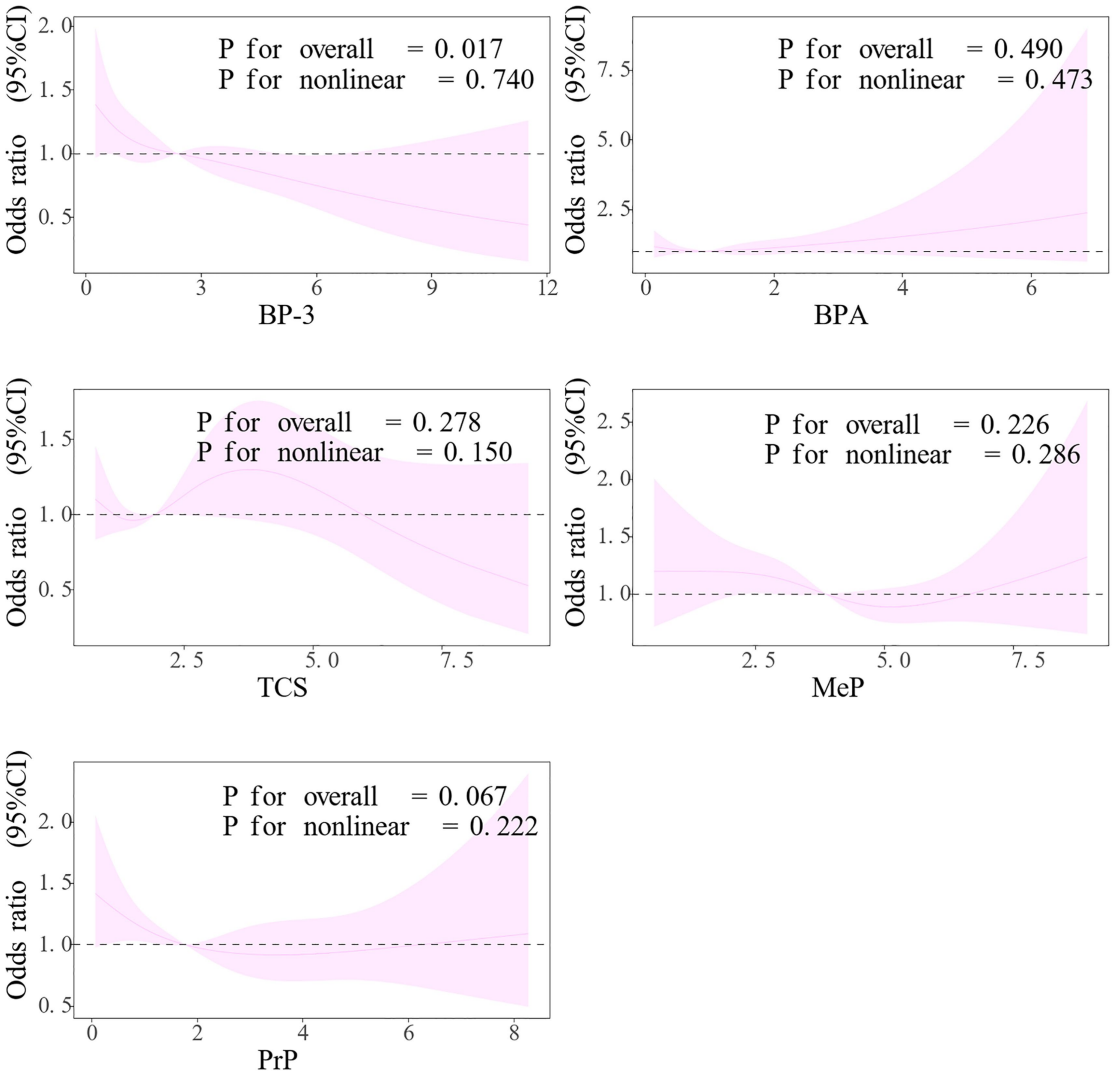
RCS Plots for the association between exposures and COPD. The model was adjusted for age, gender, race, education level, marital status, PIR, drinking, physical activity, BMI, diabetes, and hypertension. BP-3, Benzophenone-3; BPA, Bisphenol A; TCS, Triclosan; MeP, Methyl paraben; PrP, Propyl paraben.

RCS plots were constructed on the basis of adjusted linear regression models for exploring the link of exposures to COPD (Figure 4). The results revealed that BP-3 and PrP were significantly related to the outcome, while no marked nonlinear trends were observed for these two variables (BP-3: *p* for overall = 0.017, *p* for nonlinear = 0.740; PrP: *p* for overall = 0.067, *p* for nonlinear = 0.222). As the levels of these variables increased, the risk (*OR*) associated with the outcome significantly decreased. Other variables did not exhibit significant associations or nonlinear trends, indicating that their relationship with the outcome was not apparent.

### 3.4 Influence of different population characteristics on the correlation between exposure concentrations and COPD prevalence

**Figure 5:**
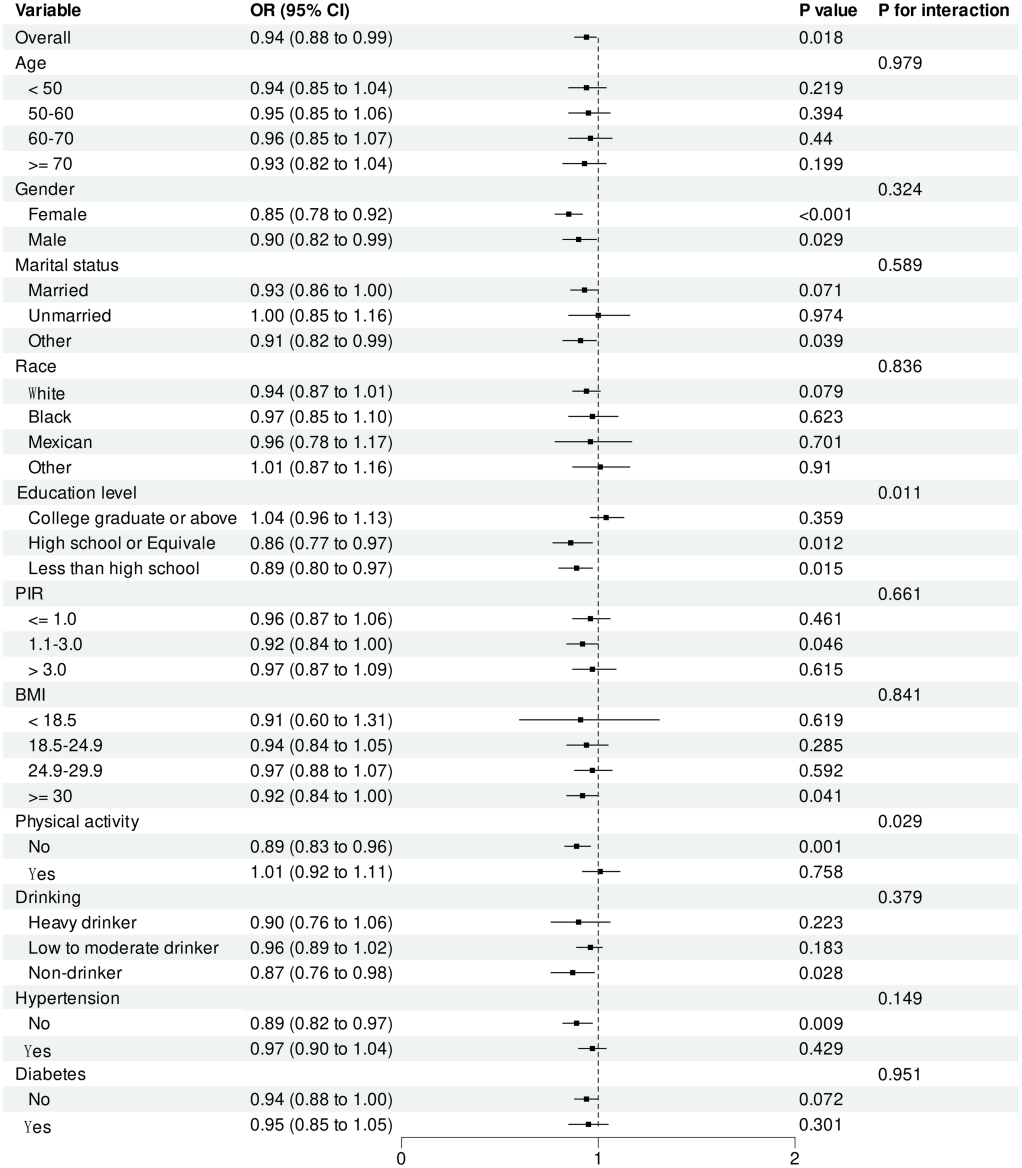
Subgroup Analysis of PrP and COPD.

**Figure 6:**
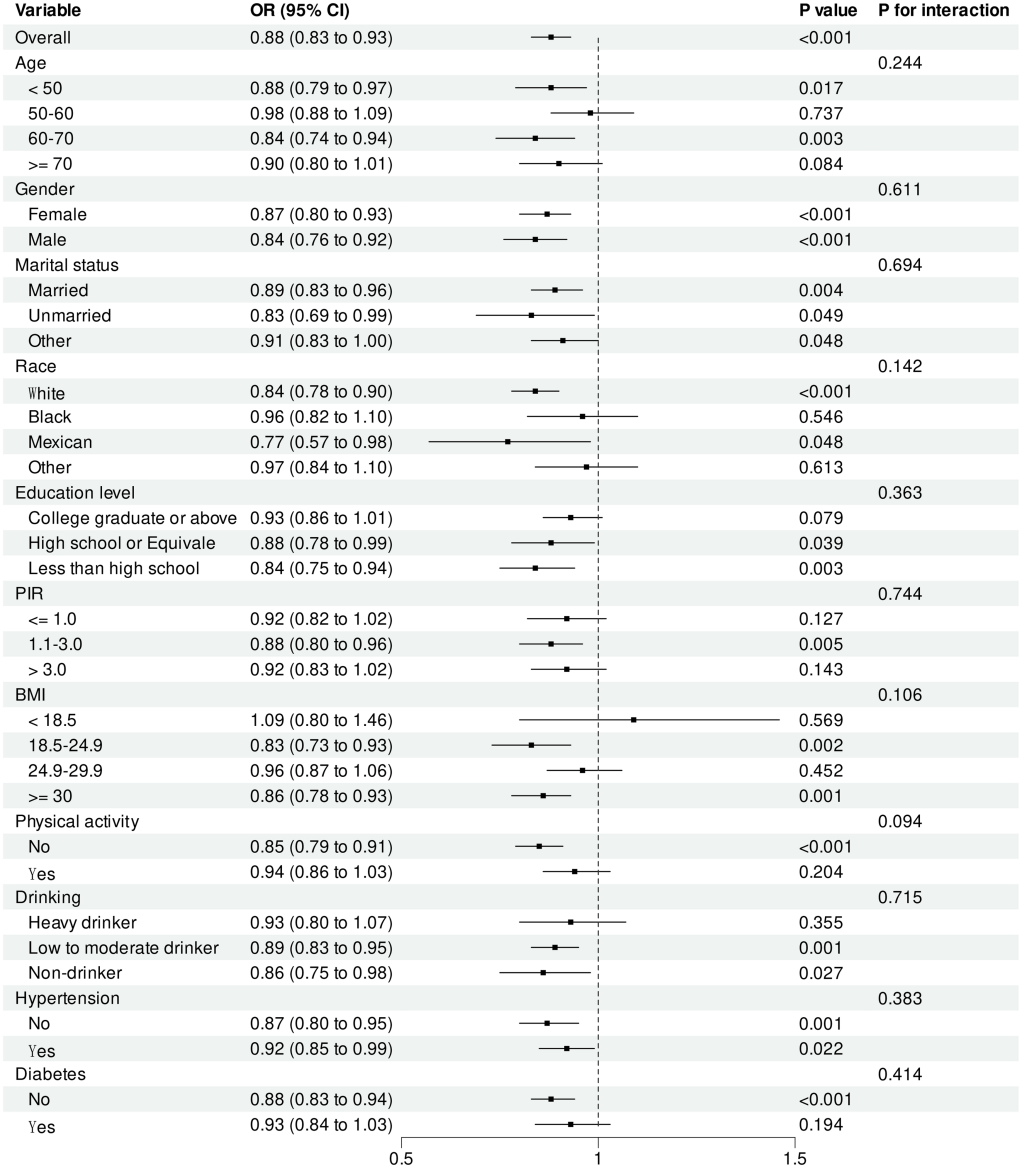
Subgroup Analysis of BP-3 and COPD.

We performed subgroup analyses to examine the influence of age, gender, race, marital status, education level, physical activity and drinking, PIR, BMI, diabetes, and hypertension on the connection between environmental exposure concentrations and COPD prevalence (Figures 5, 6). An increase in PrP concentration was demonstrated to be correlated with a reduced risk of COPD among high school graduates or equivalent and people with an education level below high school (*p* = 0.011). Moreover, an elevation in PrP concentration significantly reduced the risk of COPD among participants not engaged in labor (*p* = 0.029). No statistically significant interaction effects were noted for BP-3 concentration concerning age, gender, race, education level, drinking, physical activity, PIR, diabetes, hypertension, BMI, or physical activity (all *p* > 0.05).

### 3.5 Mediating effects of inflammatory markers on the association between exposures and COPD risk

Mediation analyses unraveled the mediating role of inflammatory markers in the correlation between exposures and COPD risk. WBC, NLR, and SII exhibited significant mediating effects on the correlation between BP-3 and COPD risk (all *p* < 0.05), with mediation proportions of 7, 4, and 2%, respectively. Additionally, WBC and NLR demonstrated notable mediating effects on the association between PrP and COPD risk, with mediation proportions of 17 and 19%, respectively (Table 3).

**Table 3:** Mediating effect of inflammation on the relationship between exposures and COPD.

		PrP				BP-3			
		Estimate	lower	upper	*p*-value	Estimate	lower	upper	*p*-value
WBC	Direct effect	−0.025	−0.053	0.003	0.079	−0.056	−0.084	−0.027	*p* < 0.01
	Indirect effect	−0.006	−0.009	−0.003	*p* < 0.01	−0.004	−0.007	−0.002	0.002
	Total effect	−0.031	−0.059	−0.003	0.030	−0.060	−0.089	−0.032	*p* < 0.01
NLR	Direct effect	−0.026	−0.054	0.002	0.071	−0.058	−0.086	−0.029	*p* < 0.01
	Indirect effect	−0.005	−0.008	−0.002	*p* < 0.01	−0.003	−0.005	0.000	0.044
	Total effect	−0.031	−0.059	−0.003	0.031	−0.060	−0.089	−0.032	*p* < 0.01
SII	Direct effect	−0.029	−0.057	−0.001	0.044	−0.058	−0.086	−0.029	*p* < 0.01
	Indirect effect	−0.002	−0.005	0.000	0.061	−0.002	−0.005	0.000	0.041
	Total effect	−0.031	−0.059	−0.003	0.031	−0.060	−0.089	−0.032	*p* < 0.01

WBC, white blood cell; NLR, Neutrophil-to-Lymphocyte Ratio; SII, systemic immune-inflammation index; BP-3, Benzophenone-3; PrP, Propyl paraben.

## 4 Discussion

Our study is the first to explore the association between environmental exposures to phenols and parabens and COPD among smokers based on NHANES data, and to elucidate the mediating effects of inflammatory markers on their relationship between these exposures and COPD risk through mediation analysis. Results indicate the following: (1) both single and mixed exposure models prove the strong correlation between BP-3, PrP, and COPD risk; (2) BP-3 and PrP are inversely linked to COPD in Americans; (3) inflammatory markers, specifically WBC and NLR, were identified as significant mediators in the negative correlation between BP-3, PrP, and COPD risk.

Over the past decades, potential EDCs such as BPA, PrP, and BP-3 have attracted considerable attention. Nearly all children and adults are exposed to these chemicals because they are widely added to personal care products, and some have been implicated in altered lung function (11, 26). We also found that COPD was more prevalent among females than males, which correlates with the increased use of the aforementioned products like sunscreen (27).

Previous multivariable logistic and linear regression analyses have demonstrated the associations between COPD and various environmental chemicals, such as ethylene oxide (28), polycyclic aromatic hydrocarbons (29), and perfluoroalkyl substances (30). These analyses typically focus on single chemicals or groups of similar chemicals, with their results relatively straightforward to interpret. However, to explore causality, it is essential to take into account mixed environmental exposures and their complex nonlinear interactions. We tested seven common chemical exposures, and contrary to prior research, our study revealed no positive correlation between low-molecular-weight chemical exposures and atopic diseases. Through single-chemical analysis, we observed a strong correlation between MeP and PrP exposure, with BP-3 or PrP being inversely associated with COPD risk among smokers, suggesting the possible confounding effects of another chemical, which may not be identified through simple models. Further analysis through WQS and Bayesian kernel machine regression models proved the association between BP-3 and PrP with COPD among smokers, with these chemicals exhibiting a strong link to COPD risk, while other exposures suggested no correlation with COPD. Subgroup analysis indicated that increased PrP concentration could reduce the risk of COPD among smokers with a high school education or less and those not engaged in labor. Other longitudinal studies have similarly revealed no notable connection between low-molecular-weight chemical exposures and respiratory disease risk: Berger et al. (31) found no significant relationship between prenatal maternal urinary biomarkers of chemical exposure and respiration-related outcomes in children, with PrP concentration related to a lower likelihood of probable asthma. Wang et al. (32) identified dust exposure and smoking as obvious risk factors for COPD in workers, but significant interaction was not noted between them.

Moreover, existing research has proved the correlation between inflammation and COPD. Yang et al. (7) proposed that the inflammatory cell profile in the sputum of COPD patients is similar, predominantly consisting of neutrophils and macrophages, with an increase in innate lymphocytes as well. Huang et al. (33) found that COPD model mice exhibit increased inflammatory infiltration, reduced differentiation of goblet cells, and enhanced secretion of inflammatory cytokines by macrophages. Benjamin et al. (34) demonstrated that a brief period of inflammation during the saccular stage of lung development disrupts elastic fiber assembly, resulting in permanent lung function impairment and the development of a COPD-like lung phenotype. However, neutrophil depletion prevents the disruption of elastic fiber assembly and restores normal lung development. Based on these findings, we investigated potential mediating effects of inflammation on the association between exposures and COPD risk through subgroup analysis, and revealed that inflammatory markers WBC and NLR significantly regulated the connection between BP-3, PrP, and COPD. This provides fresh insights into the potential mechanism underlying COPD.

However, owing to observational nature of human studies, the causal relationships of these associations remain unraveled, and inconsistencies exist across different studies. Parks et al. (35) observed that urinary BP-3 concentrations were positively related to antinuclear antibodies (ANA) in winter samples, while the correlation appeared to be reversed in summer samples. Moreover, other phenols and parabens did not exhibit a strong correlation with BP-3 and ANA overall or seasonally. This suggests that randomness, confounding factors, and interactions with other unmeasured factors could influence the findings.

The limitations of this study include the failure to address chemical reactions between different chemicals and variability in COPD risk associated with long-term exposure, despite controlling for potential confounders. It remains challenging to exclude all confounding factors. Susceptible animal models might help understand the potential impact of BP-3 and PrP on COPD pathogenesis. Furthermore, as the study relies primarily on NHANES data, and the available sample size may not accurately reflect the actual levels. Additionally, the causality between inflammatory factors, environmental exposures, and COPD cannot be established. Therefore, caution should be exercised in the interpretation of our conclusions.

Notable strengths of the study are the inclusion of the most relevant potential confounding covariates and more rigorous isolation of the association between a single chemical and atopic outcomes compared with previous studies. Subgroup and sensitivity analyses also ensure the result stability. We further delved into the mediating role of inflammatory markers among smokers, revealing that WBC and NLR partially mediated the correlation between BP-3, PrP, and COPD risk in smokers.

## 5 Conclusion

This study, based primarily on NHANES data, provides clinical evidence for the link of urinary exposures to COPD risk in smokers. BP-3 and PrP were inversely linked to the risk of developing COPD, with inflammatory markers WBC and NLR potentially serving as key mediators. These findings provide data support for exploring the specific mechanisms through which smokers develop COPD and present potential biological markers for COPD prevention and treatment.

## Data Availability

The raw data supporting the conclusions of this article will be made available by the authors, without undue reservation.
