# Supplementary material for: Association between urinary exposures and the risk of chronic obstructive pulmonary disease in smokers: results from NHANES 2007–2016
Source: Front Public Health. 2025 Apr 4;13:1548401. doi: 10.3389/fpubh.2025.1548401 (PMC12006094; doi:10.3389/fpubh.2025.1548401)
Supplement: Supplementary file 1 [file Data_Sheet_1.DOCX]

**Table S1 Distribution of exposure concentration**

| Variable | Rate (%) | LOD | Mean | Selected percentiles | | | |
| --- | --- | --- | --- | --- | --- | --- | --- |
|  |  |  |  | 25th | 50th | 75th | 95th |
| BP-3 | 94.92 | 0.4ug/L | 2.107 | 1.386 | 2.370 | 3.718 | 6.564 |
| BPA | 92.22 | 0.2ug/L | 0.866 | 0.588 | 0.956 | 1.435 | 2.411 |
| TCS | 70.60 | 1.7ug/L | 1.906 | 0.789 | 1.932 | 3.447 | 6.078 |
| BuP | 30.15 | 0.1ug/L | 0.179 | 0.068 | 0.131 | 0.182 | 2.320 |
| EtP | 49.96 | 1.0ug/L | 1.023 | 0.535 | 0.535 | 1.988 | 4.452 |
| MeP | 99.17 | 1.0ug/L | 3.537 | 2.595 | 3.863 | 5.242 | 6.859 |
| PrP | 93.86 | 0.11ug/L | 1.378 | 0.642 | 1.775 | 3.632 | 5.533 |

LOD, Limit of detection; BP-3, Benzophenone-3; BPA, Bisphenol A; TCS, Triclosan; BuP, Butyl paraben; EtP, Ethyl paraben; MeP, Methyl paraben; PrP, Propyl paraben


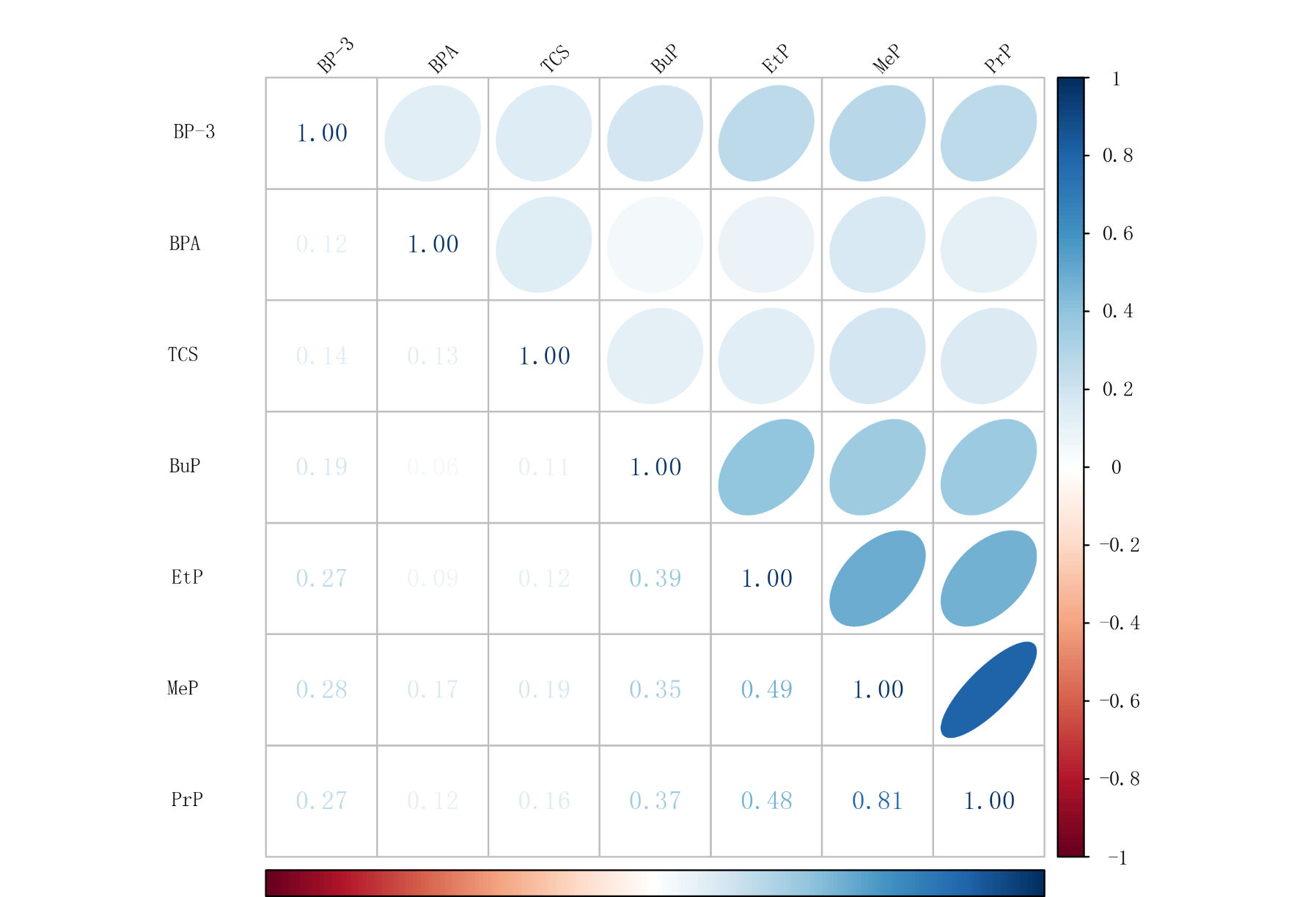


**Figure S1. Pearson correlation coefficients between exposures**

BP-3, Benzophenone-3; BPA, Bisphenol A; TCS, Triclosan; BuP, Butyl paraben; EtP, Ethyl paraben; MeP, Methyl paraben; PrP, Propyl paraben
